# Synthesis and biological evaluation of propargyl acetate derivatives as anti-mycobacterial agents

**DOI:** 10.1186/2008-2231-20-90

**Published:** 2012-12-11

**Authors:** Parisa Azerang, Ali Hossein Rezayan, Soroush Sardari, Farzad Kobarfard, Mitra Bayat, Kimia Tabib

**Affiliations:** 1 Drug Design and Bioinformatics Unit, Medical Biotechnology Department, Biotechnology Research Center, Pasteur Institute, Tehran, Iran 13164; 2 Department of Life Science Engineering, Faculty of New Science and Technology, University of Tehran, Tehran, Iran; 3 Department of Medicinal Chemistry, School of Pharmacy, Shahid Beheshti University of Medical Sciences, Tehran, Iran; 4 Phytochemistry Research Centre, Shahid Beheshti University of Medical Sciences, Tehran, Iran

**Keywords:** *Mycobacterium tuberculosis*, BCG, Propargyl alcohol, Acetylenic compounds (propargyl acetate derivatives)

## Abstract

**Background:**

The emergence of multidrug-resistant strains of *Mycobacterium tuberculosis* (Mtb) has intensified efforts to discover novel drugs for tuberculosis (TB) treatment. Targeting the persistent state of Mtb, a condition in which Mtb is resistant to conventional drug therapies, is of particular interest.

**Methods:**

This study is focused on propargyl acetate derivatives. Eight molecules were designed based on propargyl alcohols and different acid anhydrides.

**Results:**

All the synthesized compounds and commercially available ones were evaluated for anti-tuberculosis activity.

**Conclusions:**

Inhibitors against Mtb have been identified and characterized for further development into potential novel anti-tubercular drugs.

## Background

Tuberculosis (TB) is a deadly infectious disease and is the leading cause of death worldwide, killing around 2 million people annually, primarily in developing countries. The World Health Organization (WHO) estimates that over one third of the world’s population is infected with TB with approximately 8 million new cases of infection diagnosed every year [1-3]. After human immunodeficiency virus (HIV)/AIDS, TB is the second most common cause of death due to an infectious disease, and current trends suggest that TB will still be among the 10 leading causes of global disease burden in the year 2020 [4]. TB incidence is also on the rise because of the correspondingly high HIV infection rates. These two diseases progress at faster rates in co-infected individuals. The immune systems have been compromised by HIV/AIDS, so individuals fall victims to TB which takes opportunity of their weakened immune systems. There is a great interest in the scientific field to come up with a new drug(s) to combat TB. Tuberculosis is an airborne infectious disease caused by mycobacteria, mainly Mycobacterium tuberculosis (Mtb) [5]. The success of mycobacteria in producing disease relies entirely on its ability to utilize macrophages for its replication and more importantly, the maintenance of viability of host macrophages that sustain mycobacteria.

In the present scenario, due to the emergence of multi-drug resistant tuberculosis (MDR-TB) and association between HIV and TB, Directly Observed Treatment Short course (DOTS) is becoming rapidly ineffective in controlling tuberculosis. Recent reports indicate that, areas where there is a high incidence of MDR-TB, DOTS is failing to control the disease [6]. In such circumstances, the second line drugs are prescribed in combination with DOTS. However, this combination of drugs is very expensive, are less effective and thus has to be administered for a longer duration (e.g. p-amino salicylic acid), has significant side effects (e.g., cycloserine) and some are unavailable in many developing countries (e.g. fluoroquinolones).

Hence, it is clear that there is an urgent need to develop novel anti-TB drugs with improved properties such as enhanced activity against multidrug-resistance, reduced toxicity, shortened duration of therapy, rapid mycobactericidial mechanism of action, ability to penetrate host cells and exert anti-mycobacterial effect in the intracellular environment.

Alkynes or acetylenic compounds play an important role as building blocks in many synthetic transformations, and in new materials. In addition acetylenic group is common structural motifs found in various natural products and also great interest in medicinal chemistry and the pharmaceutical industry [7]. It moreover functions as a key pharmacophoric unit in acetylenic antibiotics [8] and its presence in anticancer [9] and anti-tubercular [10] agents is noteworthy.

Due to above-mentioned reasons and as a part of our ongoing research program on the synthetic methods in organic compounds [11,12], and also our drug discovery program [13,14], here a series of new acetylenic compounds (propargyl acetate derivatives ) were synthesized and evaluated for anti-tubercular activity.

## Methods

### General

Infrared spectra were determined with a Perkin-Elmer 843 spectrometer (USA). Proton nuclear magnetic resonance (^1^H NMR) spectra and carbon nuclear magnetic resonance (^13^C NMR) spectra were determined on a Bruker Avance DRX 500 MHz spectrometer (Germany) and chemical shifts are reported as δ (ppm) in CDCl_3_ solution (0.05% v/v TMS). GC/MS spectra were obtained using Agilent 7000 Triple Quad (USA). (EI at 70 eV). The chemicals used in this work were purchased from Merck (Germany), Fluka (Germany) and Sigma-Aldrich (USA) Chemical Companies.

### In vitro evaluation of anti-mycobacterial activity

*In vitro* anti-mycobacterial activity evaluations of the compounds were done by the broth microtiter dilution method) against BCG (1173P2) and ethambutol were used as standard controls.

The test compounds were initially dissolved in DMSO to give a concentration of 1 or 2 mg/L. All wells of micro plates received 100 μL of freshly prepared Middle broke 7H9 medium (Himedia, India), except first column. 200 μL of distilled water was added to the first column of 96 well plates to minimize evaporation of the medium in the test wells during incubation. Then 100 μL of test compounds with desired concentrations (1000 or 2000 μL) were added to the wells of the first row (each concentration was assayed in duplicate) and serial dilution was made from the first row to the last. Microbial suspension of BCG (1173P2) (100 μL), which had been prepared with standard concentration of 0.5 Mcfarland and diluted with 1:10 proportion by the distilled water, was added to all test wells. Plates were then sealed and incubated for 4 days at 37°C. After that 12 μl Tween 80 10% and 20 μl Alamar blue 0.01% (Himedia, India) were added to each test well. The results were assessed after 24 and 48 hours. A blue color was interpreted as no bacterial growth, and color change to pink was scored as bacterial growth. Wells with a well-defined pink color were scored as positive for growth. The MIC (minimum inhibitory concentration) was defined as the lowest drug concentration, which prevented a color change from blue to pink. Ethambutol (Irandaru, Tehran) were used as positive control and DMSO as negative control [15].

### General procedure for the preparation of propargyl acetate derivatives 3a-h

To a magnetically stirred solution of anhydride (1.2 mmol) was added 3 drops of concentrated sulfuric acid and mix thoroughly holding the large test tube containing the acetic anhydride in cold water. Then, the alcohol (1 mmol) was added to it in several increments and the reaction mixture stirred at room temperature. After, the reaction mixture was placed in hot water at about 70°C to complete the reaction; the progress of the reaction was monitored by TLC (ethyl acetate/*n*-hexane, 2:1). The purification of product was done according to the literature [16].

Prop-2-ynyl acetate (3a): Yellow liquid (yield 65%). ^1^H NMR (500 MHz, CDCl_3_): δ_H_ (ppm) 2.07 (3H, s, CH_3_), 2.46 (1H, t, ^*4*^*J*_*H*H_ = 2.5 Hz, ≡CH), 4.64 (1H,d, ^4^J_HH_ = 2.5 Hz, OCH_2_). ^13^C NMR (125 MHz, CDCl_3_): δ_C_ (ppm) 30.7 (CH_3_), 51.9 (OCH_2_), 74.8 and 77.6 (two acetylenic carbons), 170.1 (C = O). IR (KBr) (ν_max_,cm^-1^): 3468, 3296, 2948, 2125, 1733. GC EI-MS, m/z: 99 (M^+·^ + H^+^, 3%).

But-3-yn-2-yl acetate (3b): Yellow liquid (yield 70%). ^1^H NMR (500 MHz, CDCl_3_): δ_H_ (ppm) 1.49 (3H, d, CH_3_), 2.06 (3H, s, CH_3_), 2.44 (1H, s, ≡CH), 5.39-5.43 (1H, m, OCH). ^13^C NMR (125 MHz, CDCl_3_): δ_C_ (ppm) 21.00 (CH_3_), 21.2 (CH_3_), 60.0 and 82.1 (two acetylenic carbons), 72.8 (OCH), 169.8 (C = O). IR (KBr) (ν_max_,cm^-1^): 3480, 3308, 2999, 2131, 1739. GC EI-MS, m/z: 113 (M^+·^ + H^+^, 10%).

3-Methylpent-1-yn-3-yl acetate (3c): Yellow liquid (yield 63%). ^1^H NMR (500 MHz, CDCl_3_): δH (ppm) 1.02 (3H, t, ^3^*J*_*HH*_ = 7.4 Hz, CH_3_), 1.06 (3H, s, CH_3_), 1.84 (2H, q, ^3^*J*_*HH*_ = 7.4 Hz, CH_2_), 2.03 (3H, s, CH_3_), 2.54 (1H, s, ≡CH). IR (KBr) (ν_max_,cm^-1^): 3306, 2967, 2867, 2135, 1716. GC EI-MS, m/z: 140 (M^+·^, 10%).

Oct-1-yn-3-yl acetate (3d): Yellow liquid (yield 60%). ^1^H NMR (500 MHz, CDCl_3_): δH (ppm) 0.88 (3H, t, ^3^*J*_*HH*_ = 7.0 Hz, CH_3_), 1.30 (2H, m, CH_2_), 1.43 (2H, m, CH_2_), 1.75 (2H, m, CH_2_), 2.07 (3H, s, CH_3_), 2.44 (1H, d, ^*4*^*J*_*HH*_ = 2.5 Hz, ≡CH), 5.32 (H, t of d, ^3^*J*_*HH*_ = 5.0 Hz, ^*4*^*J*_*HH*_ = 2.5 Hz, OCH). ^13^C NMR (125 MHz, CDCl_3_): δ_C_ (ppm) 13.9 (CH_3_), 20.9 (CH_3_), 22.2 (CH_2_), 24.4 (CH_2_), 31.0 (CH_2_), 34.4 (CH_2_), 63.7 and 77.2 (two acetylenic carbons), 82.0 (OCH_2_), 169.9 (C = O). IR (KBr) (ν_max_,cm^-1^): 3300, 2964, 2874, 2132, 1740. GC EI-MS, m/z: 168 (M^+·^, 10%).

But-3-ynyl acetate (3e): Yellow liquid (yield 80%). ^1^H NMR (500 MHz, CDCl_3_): δ_H_ (ppm) 2.01 (1H, t, ^*4*^*J*_*HH*_ = 3.0 Hz, ≡CH), 2.10 (3H, s, CH_3_), 2.54 (2H, t of d, ^3^*J*_*HH*_ = 6.5 Hz, ^*4*^*J*_*HH*_ = 3.0 Hz, CH_2_), 4.19 (2H, t, ^3^*J*_*HH*_ = 7.0 Hz, OCH_2_).^13^C NMR (125 MHz, CDCl_3_): δ_C_ (ppm) 18.8 (CH_3_), 20.7 (CH_2_), 61.7 and 70.1 (two acetylenic carbons), 80.0 (OCH_2_), 170.8 (C = O). IR (KBr) (ν_max_,cm^-1^): 3315, 3028, 2900, 2290, 1738. GC EI-MS, m/z: 113 (M^+·^ + H^+^, 10%).

Prop-2-ynyl heptanoate (3f): Yellow liquid (yield 78%). ^1^H NMR (500 MHz, CDCl_3_): δ_H_ (ppm) 1.31 (3H, t, ^3^*J*_*HH*_ = 7.5 Hz, CH_3_), 1.63 (8H, m, 4CH_2_), 2.33 (2H, t, ^3^*J*_*HH*_ = 8.0 Hz, CH_2_), 4.45 (1H, t, ^*4*^*J*_*HH*_ = 2.0 Hz, ≡CH), 4.65 (2H, d, ^*4*^*J*_*HH*_ = 2.0 Hz, OCH_2_). ^13^C NMR (125 MHz, CDCl_3_): δ_C_ (ppm) 13.8 (CH_3_), 22.2, 24.4, 31.1, 33.8, (4CH_2_), 51.6 and 74.6, (two acetylenic carbons), 77.7 (OCH_2_), 172.9 (C = O). IR (KBr) (ν_max_,cm^-1^): 3291, 2932, 2865, 2128, 1738. GC EI-MS, m/z: 168 (M^+·^, 10%).

Oct-1-yn-3-yl heptanoate (3g): liquid (yield 55%). ^1^H NMR (500 MHz, CDCl_3_): δ_H_ (ppm) 0.89 (6H, m, 2CH_3_), 1.31 (4H, m), 1.43 (4H, m), 1.63 (4H, m), 1.75 (4H, m), 2.37 (2H, m), 2.42 (1H, d, ^*4*^*J*_*HH*_ = 2.5 Hz, ≡CH), 5.34 (1H, t of d, ^3^*J*_*HH*_ = 6.5 Hz, ^*4*^*J*_*HH*_ = 2.0 Hz, OCH). IR (KBr) (ν_max_,cm^-1^): 3310, 2963, 2872, 2127, 1736. GC EI-MS, m/z: 239 (M^+·^, 10%).

But-3-ynyl heptanoate (3h): Yellow liquid (yield 70%). ^1^H NMR (500 MHz, CDCl_3_): δ_H_ (ppm) 0.87 (3H, t, ^*3*^*J*_*HH*_ = 7.0 Hz, CH_3_), 1.30 (4H, m, CH_2_), 1.61 (2H, m, CH_2_), 1.98 (1H, t, ^*4*^*J*_*HH*_ = 2.5 Hz, ≡CH), 2.32 (2H, m, CH_2_), 2.50 (2H, t of d, ^3^*J*_*HH*_ = 7.0 Hz, ^*4*^*J*_*HH*_ = 3.0 Hz, CH_2_), 4.16 (2H, t, ^*3*^*J*_*HH*_ = 7.0 Hz, OCH_2_ ). ^13^C NMR (125 MHz, CDCl_3_): δ_C_ (ppm) 13.9 (CH_3_), 19.0, 22.3, 24.4, 31.2, 34.14 (5CH_2_), 61.9 and 69.9 (two acetylenic carbons), 80.1(OCH_2_), 173.6 (C = O). IR (KBr) (ν_max_,cm^-1^): 3304, 2961, 2868, 2126, 1735. GC EI-MS, m/z: 182 (M^+·^, 5%).

## Results and discussion

Esterification of alcohols using acid anhydrides in the presence of acid or base catalyst has been known and extensively used by organic chemists for nearly 100 years [17]. Hydroxyl compounds are often converted to acetate derivatives for protection or characterization of the structure. For this purpose, acetic anhydride is commonly employed with an acid or base catalyst, such as zinc chloride, concentrated sulphuric acid, anhydrous sodium [18] acetate or, most often, pyridine [19].

**Scheme 1:**
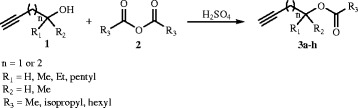
Synthesis of propargyl acetate derivatives.

In the present work, propargyl acetate derivatives 3a-h were prepared by acylation of propargyl alcohols 1 with acid anhydrides 2 in H_2_SO_4_ (Scheme 1) [20].

**Figure 1:**
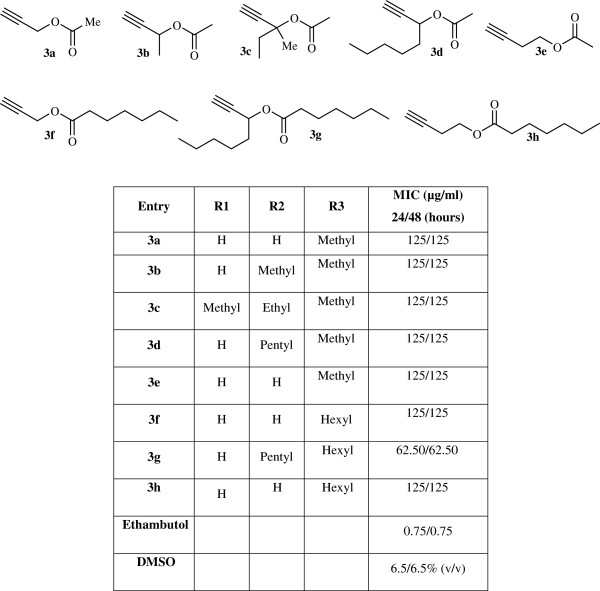
Synthesized propargyl acetate derivatives and their MIC.

The reactions are straightforward and as indicated in Scheme 1, treatment of various propagyl alcohols 1 with acid anhydrides 2 in H_2_SO_4_ led to products 3a-h. To explore the scope and variety of biological activity for products of this reaction, we have examined various primary, secondary and tertiary propagyl alcohols in the presence of aliphatic and aromatic acid anhydrides in H_2_SO_4_ at room temperature. As indicated Figure 1, the reaction proceeds efficiently and led to highly functionalized propargyl acetate derivatives 3a–h in good yields.

The structures of compounds 3a–h were deduced from their ^1^H NMR and ^13^C NMR spectroscopic data. For example, the ^1^H NMR spectrum of 3a exhibited a singlet at 2.07 (CH_3_), a triplet at 2.46 (^4^*J*_*HH*_ = 2.5Hz) for acetylenic hydrogen (≡CH) and a doublet at 4.64 (^4^*J*_*HH*_ = 2.5 Hz, OCH_2_). The ^1^H decoupled ^13^C NMR spectrum of 3a showed 5 distinct resonances, and partial assignment of these resonances is given in experimental section.

All the synthesized compounds were evaluated for anti-tubercular activity and the results are summarized in Figure 1.

**Figure 2:**
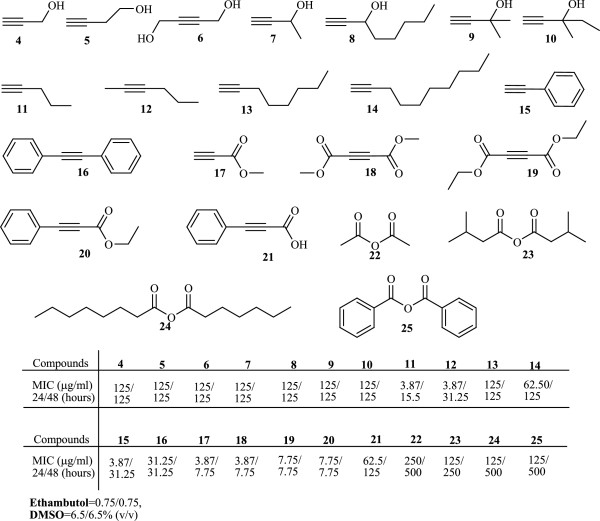
Commercially available substrates and their MIC (minimal inhibition concentration).

Compounds 3a-h have almost the same activity except 3 g that is slightly stronger; it shows that the changing of R_1_, R_2_ and R_3_ is not important in these compounds. Therefore, we did not synthesis more derivatives from these compounds. In continuation of the work, we investigated different groups of organic compounds that all of these compounds containing acetylenic group in difference situation, such as alcohols (4–10), acetylenic compounds (11–16), esters (17–20), acids (21), and anhydrides (22–25) (without acetylenic group). In these compounds (4–25), the nature and location of mentioned functional group can affect the anti-bacterial activity of acetylenic compounds. As can be seen from Figure 2, among these acetylenic compounds (11,12,15, 16) and esters (17–20), have almost similar effect and more bioactivity than others. It seems that replacement of alkyne hydrogen with an alkyl or aryl group in case of compounds 12 and 16 and a balanced hydrophobicity playing with special features, in case of compounds 11 and 15 have beneficial effect on the bioactivity. However, it is necessary to examine the possible mechanism of action in detail to clear the reason for the variations of activity.

It is interesting to note that comparison of compounds 3a-h in Figure 1 and 17–20 in Figure 2 which are ester shows that 17–20 more active than 3a-h (3.87 vs 125). This can be because of difference in structure of two classes of esters. The main structural difference between 3a-h and 17–20 is that in 17–20 the ester group is terminal whilst in 3a-h is internal. The conjugation of double bond with the triple bond and the resulting dipole moment is a feature that can explain this difference. The same effect can play a role for the better activity of compounds 15 and 16.

## Conclusions

In conclusion, the synthesized new class of propargyl acetate derivatives and commercially available compounds were evaluated for anti-tuberculosis activity. Among them, 11, 12, 15, 17 and 18 exhibited promising activity against of *M. bovis*.

## Competing interests

The authors declare that they have no competing interests.

## Authors’ contribution

First person: synthesis of the molecules, bioassay and writing some parts of the draft, second person: preparation of the draft, third person: design of the molecules and PI, fourth person: interpretation of MS and IR spectra, fifth person: obtaining MS spectra, sixth person: obtaining IR spectra. All authors read and approved the final manuscript.
